# An Adverse Outcome Pathway for Decreased Lung Function Focusing on Mechanisms of Impaired Mucociliary Clearance Following Inhalation Exposure

**DOI:** 10.3389/ftox.2021.750254

**Published:** 2021-12-14

**Authors:** Karsta Luettich, Monita Sharma, Hasmik Yepiskoposyan, Damien Breheny, Frazer J. Lowe

**Affiliations:** ^1^ Philip Morris International R&D, Philip Morris Products S.A., Neuchatel, Switzerland; ^2^ PETA Science Consortium International e.V., Stuttgart, Germany; ^3^ British American Tobacco (Investments) Ltd., Group Research and Development, Southampton, United Kingdom; ^4^ Broughton Nicotine Services, Earby, Lancashire, United Kingdom

**Keywords:** adverse outcome pathway, AOP, mucociliary clearance, ciliary beat frequency, lung function, new approach methodologies, NAMs, inhalation toxicity

## Abstract

Adverse outcome pathways (AOPs) help to organize available mechanistic information related to an adverse outcome into key events (KEs) spanning all organizational levels of a biological system(s). AOPs, therefore, aid in the biological understanding of a particular pathogenesis and also help with linking exposures to eventual toxic effects. In the regulatory context, knowledge of disease mechanisms can help design testing strategies using *in vitro* methods that can measure or predict KEs relevant to the biological effect of interest. The AOP described here evaluates the major processes known to be involved in regulating efficient mucociliary clearance (MCC) following exposures causing oxidative stress. MCC is a key aspect of the innate immune defense against airborne pathogens and inhaled chemicals and is governed by the concerted action of its functional components, the cilia and airway surface liquid (ASL). The AOP network described here consists of sequences of KEs that culminate in the modulation of ciliary beat frequency and ASL height as well as mucus viscosity and hence, impairment of MCC, which in turn leads to decreased lung function.

## 1 Introduction

Regulatory frameworks are moving towards risk assessment approaches that better protect human health and are not reliant on testing in animals. Therefore, 21st century science is incorporating the use of human-relevant methods that are ethical, scientifically sound, and can accurately predict the toxicity of chemicals. *In silico* models that consider human-relevant parameters as well as *in vitro* methods that vary in complexity—spanning from mono-to co-culture systems—are already being used to predict human outcomes. For example, the Organisation for Economic Co-operation and Development (OECD) (OECD, 2021) uses combined information from several sources (e.g. *in silico* predictions, *in chemico*, *in vitro* data) to predict pathological outcomes in humans in response to chemical exposure. Anchored to known mechanisms of human toxicity such mechanism-based approaches enable us to understand whether a chemical will be toxic and through which pathway(s) it may act to cause the adverse outcome (AO) (Clippinger et al., 2018).

Adverse outcome pathways (AOP) are a means to organize known information related to a pathological outcome and understand the mechanism leading to the adverse effect. Starting with a molecular initiating event (MIE) and ending in an AO, AOPs are a sequence of causally linked key events (KE) that span different levels of biological organization—from the molecular to the whole organism level (Ankley et al., 2010). An AOP may not necessarily include every single event that contributes to the development of the AO, but it does include all KEs that are critical for its development (OECD, 2017). *In vitro* and *in silico* assays that measure each of the KEs of an AOP can be used to design testing approaches that closely predict human responses and replace the need for *in vivo* data in order to derive benchmark values for determining the potential adverse health impacts of chemicals. When combined with existing data and physicochemical information related to the test substance, AOP-based testing can help develop integrated approaches that can predict human responses (OECD, 2021). Given the usefulness of AOPs in the risk assessment of chemicals, the OECD launched a program for the development of AOPs in 2012; called the AOP-Wiki, this program is overseen by the Extended Advisory Group on Molecular Screening and Toxicogenomics (EAGMST). Several online resources, including the OECD AOP users’ handbook, are available to aid developers in compiling AOPs on the AOPwiki (OECD, 2018). There are currently more than 300 AOPs online, at various stages of completion. One of these is AOP148 [EGFR Activation Leading to Decreased Lung Function, https://aopwiki.org/aops/148], which is extended and complemented by the AOP network described here.

With a surface area of ∼100 m^2^ and ventilated by 10,000 to 20,000 L of air per day (National Research Council, 1988; Frohlich et al., 2016), the lungs are a major barrier that protect the body from a host of external factors that enter the respiratory system and may cause lung pathologies. Mucociliary clearance (MCC) is a key aspect of the innate immune defense against airborne pathogens and inhaled particles. MCC is governed by the concerted action of its functional components, the cilia and the airway surface liquid (ASL), where the latter comprises mucus and the periciliary layer (PCL) (Bustamante-Marin and Ostrowski, 2017). Healthy subjects produce >10 ml airway secretions daily (King, 2006), which are continuously transported by the mucociliary escalator. Disturbances in any of the processes that regulate ASL volume, mucus production, mucus viscoelastic properties, or ciliary function can cause MCC dysfunction and are linked to airway diseases such as chronic obstructive pulmonary disease (COPD) and asthma, both of which bear a significant risk of increased morbidity and mortality. The mechanism by which exposure to inhaled toxicants might lead to mucus hypersecretion and thereby impact pulmonary function has already been mapped in AOP148 on decreased lung function. However, whether an exposure-related decline in lung function is solely related to excessive production of mucus is debatable, particularly in light of the close relationship between mucus, cilia function, and efficient MCC. To date, no single event has been attributed to MCC impairment. This AOP work evaluates the major processes known to be involved in ensuring efficient MCC and consists of sequences of KEs that culminate in the modulation of ASL, ciliary beat frequency (CBF), and mucus viscosity. Together, these processes impair MCC, which—when persistent—leads to decreased lung function. Evidence was gathered from the peer-reviewed literature from multiple sources (e.g., PubMed, Web of Science, Scopus) by keyword searches. No publication date limit was applied. Both empirical and quantitative evidence was captured, consolidated and transferred to the corresponding KE and KER pages on the AOPwiki following the recommendations in the AOP User’s Handbook.

## 2 Summary of Key Events and Mechanisms

**FIGURE 1:**
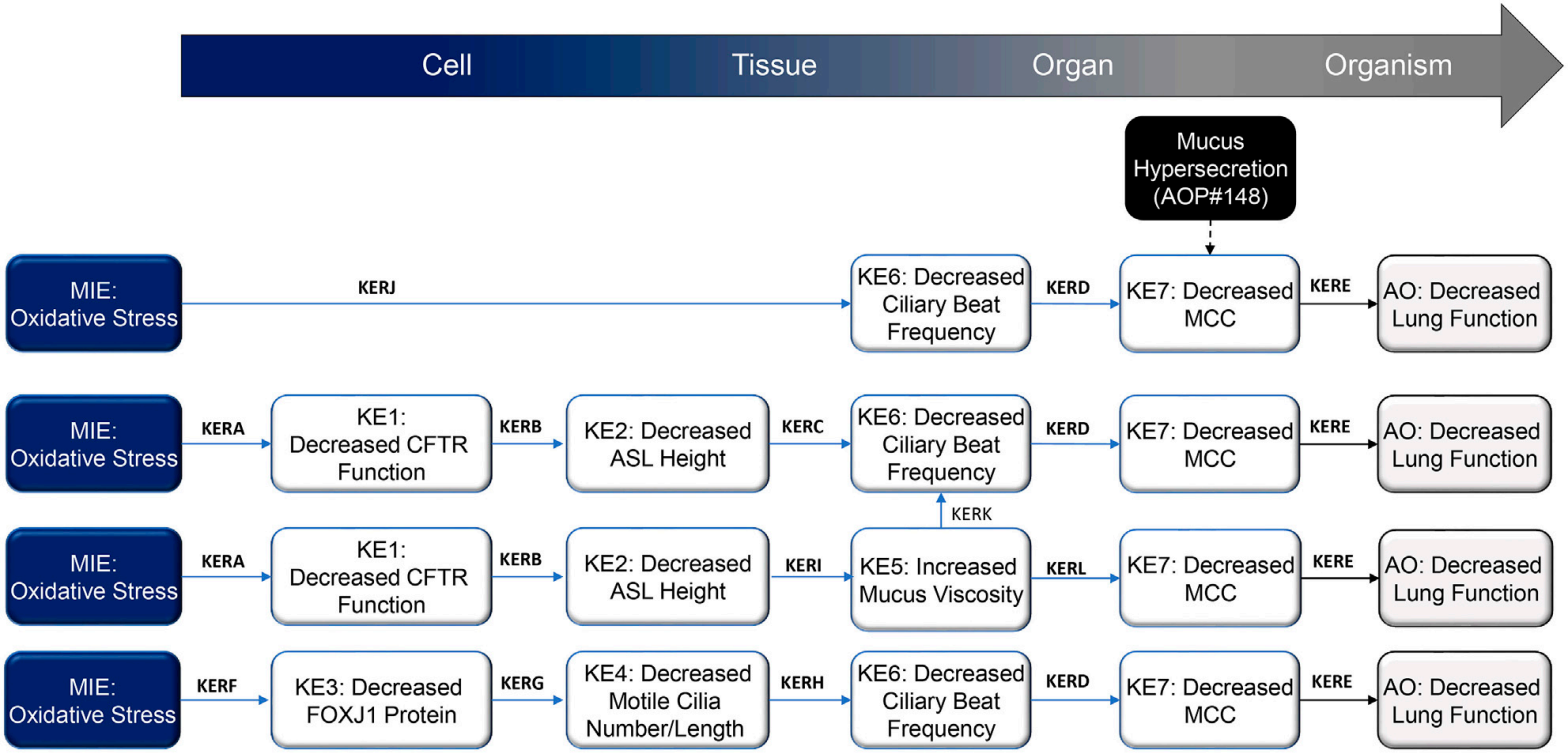
Schematic representation of the proposed decreased lung function adverse outcome pathways (AOP), focusing on mechanisms that result in impaired mucociliary clearance (MCC) following inhalation exposure. Abbreviations: AO, adverse outcome; ASL, airway surface liquid; CFTR, cystic fibrosis transmembrane regulator; FOXJ1, forkhead box protein J1; KE, key event; KER; key event relationship; MCC, mucociliary clearance; MIE, molecular initiating event.

The epithelium of the respiratory tract has a powerful defense mechanism against airborne pollutants, owing to the combined performance of mucus-producing goblet cells and ciliated cells that are covered with microtubular projections called cilia. In response to various irritants and pathogens, goblet cells produce and secrete mucus, and the cilia sweep the mucus upward through coordinated beating motions, thus clearing the airways of these substances—a process which is termed MCC. Optimal MCC is dependent on multiple factors, including cilia number and structure, ASL height^1^, and the physical and chemical properties of mucus. Any disturbances in these factors can lead to impaired MCC. A summary schematic of the AOP network delineating processes that lead from oxidative stress to decreased lung function is presented in Figure 1 and detailed on the AOPwiki (https://aopwiki.org/aops/411, https://aopwiki.org/aops/424, https://aopwiki.org/aops/425).The MIE for this network of AOP is oxidative stress. Oxidative stress is generally regarded as a redox imbalance characterized by the increased production of oxidative species and concurrent depletion of antioxidant defenses. Thus, the overall redox balance of the cell/tissue is tipped in favor of oxidation. Various highly reactive species, collectively referred to as “reactive oxygen species” (ROS) or “reactive nitrogen species” (RNS), are formed continuously at relatively low concentrations during the normal biochemical functioning of cells and tissues. They are highly unstable because they contain unpaired electrons capable of initiating oxidation reactions and include free radicals such as hydroxyl radicals, superoxide anions, oxygen radicals, nitric oxide, and non-free radicals, such as hydrogen peroxide, peroxynitrite and hypochlorous acid (Rahman et al., 2006). However, upon exposure to certain xenobiotics or in the presence of pathogens, cells may form excessive ROS/RNS, which may react with cellular components such as proteins, lipids and nuclear material, leading to the dysfunction of these components and, ultimately, cell death and disease manifestation (Halliwell and Aruoma, 1991; Berlett and Stadtman, 1997). Protective enzymes such as catalase, glutathione peroxidase, superoxide dismutase, and thioredoxin—in combination with radical scavengers such as glutathione, ascorbic acid, uric acid and vitamin E—work in concert to maintain ROS/RNS levels that are not overly damaging to cells and cellular systems (Rahman et al., 2006).

In the lungs, free radical species may be endogenously produced or introduced following exposure to exogenous sources, such as air pollutants, inhaled chemicals/therapeutics, and cigarette smoke (Church and Pryor, 1985). The main cellular sources of reactive species in the lungs include neutrophils, eosinophils, alveolar macrophages, alveolar epithelial cells, bronchial epithelial cells, and endothelial cells (Holland et al., 1990; Kinnula et al., 1992; Kinnula et al., 1995); these cells may increase their ROS/RNS production in response to infection or tissue damage. ROS/RNS generally inflict their effects by remodeling extracellular matrix and stimulating mucus secretion and repair responses (Poli and Parola, 1997). Oxidative stress can lead to a variety of respiratory diseases, such as asthma, acute respiratory distress syndrome and COPD (Rahman and MacNee, 1996; Chabot et al., 1998).With respect to this specific AOP, localized oxidative stress in the airways as a result of cigarette smoke exposure, for example, can cause damage to various proteins linked to the regulation of cilia function. Reduced expression of the *CFTR* (cystic fibrosis transmembrane conductance regulator) transcript, diminished CFTR protein levels, and altered chloride (Cl^−^) channel gating lead to acquired CFTR dysfunction (Clunes et al., 2012; Braun, 2014), which perturbs the height of the ASL and facilitates cilia collapse. Furthermore, oxidative damage has been reported to decrease the FOXJ1 (forkhead box protein J1) gene and protein expression, a critical protein involved in the assembly of motile cilia (Milara et al., 2012; Brekman et al., 2014; Garcia-Arcos et al., 2016; Valencia-Gattas et al., 2016; Ishikawa and Ito, 2017). Collectively, these perturbations result in decreased MCC from the upper airways.

CFTR is a multi-domain membrane protein belonging to the large family of adenine nucleotide-binding cassette transporters (Riordan, 2008). It is an integral membrane glycoprotein which functions as cyclic adenosine monophosphate (cAMP)-activated and phosphorylation-regulated Cl^−^ channel at the apical membrane of epithelial cells (Farinha et al., 2013). In respiratory epithelia, CFTR mediates fluid and electrolyte transport, and its function is critical to ASL homeostasis. Exposure to inhaled oxidants leads to decreased CFTR gene and protein expression as well as CFTR internalization, which reduces protein presentation at the membrane and reduces or abolishes short-circuit currents (Cantin et al., 2006a; Cantin et al., 2006b; Clunes et al., 2012; Sloane et al., 2012; Rasmussen et al., 2014). Decreased CFTR expression (KE1) in airway epithelium has been observed in cystic fibrosis and after hypoxia and cigarette smoke exposure, resulting in reduced Cl^−^ transport and, ultimately, reduced ASL depth (Alexander et al., 2012; Clunes et al., 2012; Rasmussen et al., 2014; Woodworth, 2015; Raju et al., 2016).

The ASL is a liquid layer on the apical side of the respiratory epithelium, reportedly between 5 and 100 μm in depth (Widdicombe and Widdicombe, 1995). It consists of an inner aqueous PCL, which spans the length of the cilia, and an outer gel-like mucus layer. The PCL has a low viscosity and enables ciliary beating, thereby facilitating the movement of the outer mucus layer toward the glottis and, ultimately, its removal by cough or ingestion (Antunes and Cohen, 2007). Both ASL composition and height are considered critical for its function (Fischer and Widdicombe, 2006). Under physiological conditions, ASL composition and height are regulated through vectorial transport of electrolytes, driven by transepithelial transport and apical secretion of Cl^−^ by (predominantly) CFTR, which results in passive water secretion and, consequently, increased ASL height. Absorption of sodium ions (Na^+^) on the apical side by the epithelial sodium channel (ENaC) and its interaction with the basolateral Na^+^/K^+^-ATPase leads to net absorption of Na^+^, which in turn drives fluid absorption and therefore decreases ASL height (KE2) (Hollenhorst et al., 2011; Althaus, 2013). Impairment of CFTR or ENaC function can lead to the dysfunction of the other ion channel (increased CFTR activity leads to decreased ENaC activity and vice versa) (Hobbs et al., 2013; Munkholm and Mortensen, 2014), resulting in perturbation of ASL height.

The number, structure, and cohesive beating of the motile cilia lining the upper and lower respiratory tract are critical for efficient MCC. Motile cilia are microtubular organelles, 6–7 µm long and 0.2–0.3 µm in diameter (Brooks and Wallingford, 2014; Yaghi and Dolovich, 2016). They protrude from the cell surface and generate directional flow of fluid though coordinated beating. Approximately 50–80% of the human respiratory epithelium is comprised of ciliated cells; each ciliated cell is covered by more than a hundred motile cilia, which move mucus upwards (together with mucus-trapped substances) upward (Yaghi and Dolovich, 2016; Bustamante-Marin and Ostrowski, 2017). Cilia formation is initiated and coordinated by a distinct gene expression program, led by the transcription factor FOXJ1 (Brody et al., 2000; Zhou and Roy, 2015). The multiple motile cilia assembly factors MCIDAS (multiciliate differentiation and DNA synthesis associated cell cycle protein) and GMNC (geminin coiled-coil domain containing) converge in positively regulating FOXJ1 (Stubbs et al., 2012; Arbi et al., 2016; Berta et al., 2016), whereas NOTCH (Notch homolog (Drosophila))-, IL-13 (interleukin-13)- or EGF (epidermal growth factor)-triggered signaling antagonizes FOXJ1-driven multiciliogenesis (Gomperts et al., 2007; Shaykhiev et al., 2013; Gerovac et al., 2014; Gerovac and Fregien, 2016). Although various other factors are involved in multiple motile cilia assembly—including MYB (MYB proto-oncogene), RFX3 (regulatory factor X3), ULK4 (Unc-51 like kinase 4), Wnt signaling, and others—they mostly act upstream or in parallel to FOXJ1 (Tan et al., 2013; Choksi et al., 2014; Liu et al., 2016; Schmid et al., 2017). FOXJ1 appears to be the major factor in multiciliogenesis, whereby its activity is necessary and also sufficient for programming cells to assemble functional motile cilia (Vij et al., 2012; Zhou and Roy, 2015). It is not surprising, therefore, that a decrease in FOXJ1 levels (KE3) inhibits ciliogenesis in multiciliated cells in zebrafish and *Xenopus* (Stubbs et al., 2008), and knockdown of FOXJ1 results in almost complete absence of cilia in mouse epithelial cells (Chen et al., 1998; Brody et al., 2000). FOXJ1 expression also decreases in cigarette smoke extract-treated human airway epithelial cells, leading to suppression of cilia growth, which can be restored by overexpression of the protein (Brekman et al., 2014).

Because ciliated cell density and the multiple motile cilia length and number per cell correlate with CBF—which is routinely used as a predictor of MCC efficiency (King, 2006)—it follows that, if cilia numbers decrease (KE4), CBF decreases (KE6). Cohesive beating of multiple motile cilia with a specific frequency and pattern propels mucus (and trapped particles or pathogens) upwards, creating a continuous movement (Chilvers and O'Callaghan, 2000). CBF is influenced by several factors, including structural modulation in the cilia and the concentrations of the cyclic nucleotides cAMP and cGMP and intracellular calcium (Ca^2+^) (Rubin, 2002). CBF also depends on the physical and chemical properties of the ASL. If ASL height decreases following, for example, exposure to cigarette smoke, the cilia cannot extend to their full height, and MCC efficiency will drop. In addition, reduced ASL height results in airway dehydration, which increases mucus viscosity (KE5) (Gheber et al., 1998; Lai et al., 2009; Fahy and Dickey, 2010). Increased mucus viscosity, in turn, decreases CBF and slows the transport of mucus on the mucociliary escalator (i.e., decreases MCC; KE7). In chronic inflammatory states, as seen (for example) in the lungs of cystic fibrosis, asthma, or COPD patients, decreased MCC can lead to mucus impaction, resulting in the formation of mucus plugs, which then in turn obstruct the airways and, consequently, lead to decreased lung function (AO) over time (Wanner et al., 1996; Szczesniak et al., 2017; Dunican et al., 2021).

## 3 Empirical Evidence for Key Event Relationships

Table 1 presents a summary of supporting evidence for each of the KERs in this AOP. KERs are rated as “strong”, “moderate”, or “weak” on the basis of empirical evidence supporting a change in an upstream KE (KEup) leading to an appropriate change in the immediate downstream KE (KEdown). Other considerations are whether KEups occur at lower doses, earlier time points, and at a higher incidence than KEdowns and if there are any inconsistences in the published data. The experimental evidence for a causal relationship between the KEup and KEdown in this AOP has been provisionally rated as “moderate” or “strong” in most cases.

**TABLE 1:** Empirical evidence for key event relationships (KER).

KER	Defining question: Does empirical evidence support that a change in KEup leads to an appropriate change in KEdown? Does KEup occur at lower doses, earlier time points, and higher in incidence than KEdown? Inconsistencies?	
	High (Strong): Multiple studies showing dependent change in both events following exposure to a wide range of specific stressors. No or few critical data gaps or conflicting data	
	Moderate: Demonstrated dependent change in both events following exposure to a small number of stressors. Some inconsistencies with expected pattern that can be explained by various factors	
	Low (Weak): Limited or no studies reporting dependent change in both events following exposure to a specific stressor, and/or significant inconsistencies in empirical support across taxa and species	
KERA	Strong	Inducers of oxidative stress such as cigarette smoke reduced CFTR expression at both the RNA Cantin et al. (2006a); Cantin et al. (2006b); Qu et al. (2009); Rennolds et al. (2010) and protein (Cantin et al. (2006b); Qu et al. (2009); Rennolds et al. (2010); Sloane et al. (2012); Hassan et al. (2014); Rasmussen et al. (2014); Xu et al. (2015) level *in vitro*. CFTR protein expression was lower in the airways of smokers compared to non-smokers Dransfield et al. (2013). In some of these studies, an accompanying decrease in Cl^−^ conductance was also observed Qu et al. (2009); Rennolds et al. (2010); Sloane et al. (2012). There are many studies that support a direct link between oxidative stress and decreased CFTR function *in vitro*, *ex vivo*, *in vivo* and in human subjects. Human primary epithelial cells and cell lines of respiratory epithelial origin have consistently decreased conductance of Cl^−^ and other ions following exposure to cigarette smoke and other oxidants (Cantin et al. (2006b); Schwarzer et al. (2008); Raju et al. (2013); Lambert et al. (2014); Schmid et al. (2015); Raju et al. (2016); Chinnapaiyan et al. (2018), which could be reversed upon antioxidant treatment Raju et al. (2013); Lambert et al. (2014); Schmid et al. (2015). Similar observations were made under hypoxic conditions Brézillon et al. (1997); Zhang et al. (2013); Woodworth, (2015). Antioxidants could also increase Cl^−^ conductance and anion transport in the absence of oxidant treatment or hypoxia induction in human and murine respiratory cells *in vitro* and in *ex vivo* tissues Azbell et al. (2010); Alexander et al. (2011); Conger et al. (2013). Healthy smokers and smokers with COPD have reduced Cl^−^ conductance Sloane et al. (2012); Dransfield et al. (2013) and increased sweat chloride concentrations Raju et al. (2013); Courville et al. (2014)
Oxidative stress leading to decreased CFTR function		
KERB	Strong	As a major Cl^−^ channel in the respiratory epithelium, CFTR levels and function are vital for maintenance of ASL homeostasis. *In vitro* studies on the effects of cigarette smoke exposure on human lung primary cells and cell lines showed a reduction in ASL height, associated with decreased CFTR levels Hassan et al. (2014); Rasmussen et al. (2014); Xu et al. (2015); Ghosh et al. (2017) and decreased Cl^−^ current Lambert et al. (2014); Raju et al. (2016). Moreover, pharmaceutical stimulation and inhibition of CFTR function and expression directly increased and decreased ASL height, respectively Song et al. (2009); Van Goor et al. (2009); Van Goor et al. (2011); Tuggle et al. (2014)
Decreased CFTR function leading to decreased ASL height		
KERC	Weak	Concurrent ASL height and CBF decreases were noted in human 3D airway epithelial cultures following exposure to cigarette smoke Åstrand et al. (2014); Xu et al. (2015) and following the addition of large dextran molecules, low-melting point agarose or endogenous mucus Button et al. (2012). Treatment of human airway epithelial with an ENaC inhibitor prevented the cigarette smoke effect on ASL height and CBF Åstrand et al. (2014). In addition, treatment of cystic fibrosis airway cultures with a CFTR-modifying drug increased both ASL height and CBF Van Goor et al. (2009)
Decreased ASL height leading to decreased CBF		
KERD	Moderate	A decrease in CBF resulting from sulfur dioxide exposure reduced mucociliary clearance in dogs Yeates et al. (1997) and mucociliary activity in guinea pig tracheas Knorst et al. (1994). In rats, formaldehyde inhalation exposure resulted in lower numbers of ciliated cells, while ciliary activity and mucus flow rates were decreased in a dose and time-dependent manner (Morgan et al. (1986). In humans, CBF positively correlates with nasal mucociliary clearance time Ho et al. (2001), and bronchiectasis patients have lower nasal CBF and slower mucociliary transport (MCT) Rutland and Cole, (1981). Administration of nebulized CBF inhibitors and enhancers quantifiably decreased or increased mucociliary clearance, respectively Boek et al. (1999); Boek et al. (2002). Increased CBF and MCT was also noted in human sinonasal epithelial cell cultures treated with Myrtol^®^, an essential oil distillate Lai et al. (2014) and in sheep tracheas and human airway epithelial cultures subjected to temperature changes Kilgour et al. (2004); Sears et al. (2015). Exposures of frog palate epithelia to formaldehyde and PM10 reduced MCC and mucociliary transport, but only formaldehyde-treated epithelia showed decreases in CBF Morgan et al. (1984); Macchione et al. (1999); Fló-Neyret et al. (2001)
Decreased CBF leading to decreased MCC		*Ex vivo* treatment of sheep trachea with acetylcholine and epinephrine increased CBF, but only acetylcholine increased surface liquid velocity, while both parameters were decreased upon incubation with platelet-activating factor Seybold et al. (1990)
KERE	Moderate	Changes in MCC rate are typically paralleled by effects on lung function in several studies where both endpoints have been assessed. In patients with primary ciliary dyskinesia, absence of cilia motion prevents normal MCC and consequently, lung function is reduced Denizoglu Kulli et al. (2020). In cystic fibrosis patients, the ASL is depleted resulting in impaired MCC Boucher, (2004). Although the known CFTR genotypes can result in a variety of phenotypes Derichs, (2013), clinical data indicate that some specific gene defects, such as the p.Phe508del variant, are more frequently associated with decreased lung function indices (e.g. FEV1% predicted, FVC % predicted, FEF25-75) Kerem et al. (1990); Johansen et al. (1991); Schaedel et al. (2002). Both cigarette smoking and occupational exposure to biomass fumes led to slower MCC and reduced FEV1% predicted and FEV1/FVC Ferreira et al. (2018). Nasomucociliary clearance was slower in COPD smokers compared to former smokers with COPD or to nonsmokers Ito et al. (2015). Allergen challenge in asthma patients resulted in both reduced MCC and FEV1, which could be reversed by inhalation of hypertonic saline solution Alexis et al. (2017). In cystic fibrosis patients, treatment with mucolytic agents Laube et al. (1996); McCoy et al. (1996); Quan et al. (2001); Elkins et al. (2006); Amin et al. (2011); Donaldson et al. (2018) or a CFTR potentiator Rowe et al. (2014) improved both MCC and lung function (FEV1, FVC and FEF25-75)
Decreased MCC leading to decreased lung function		
KERF	Moderate	Cigarette smoke-induced oxidative stress downregulates FOXJ1 expression at both the gene and protein levels in human lung cells *in vitro* Milara et al. (2012); Brekman et al. (2014); Valencia-Gattas et al. (2016); Ishikawa and Ito, (2017). Oxidative stress induced by human respiratory syncytial virus reduces FOXJ1 mRNA levels, which can be restored by treatment with antioxidants or the phosphodiesterase 4 inhibitor roflumilast N-oxide Akaike et al. (1990); Geiler et al. (2010); Mata et al. (2012). In mice, thoracic irradiation results in free radical generation and subsequent reduction in FOXJ1 mRNA expression Bernard et al. (2012). Many genes that are transcriptionally regulated by FOXJ1 are also downregulated following exposure to cigarette smoke, which implies a reduction in FOXJ1 transcriptional activity Brekman et al. (2014)
Oxidative stress leading to decreased FOXJ1 protein		
KERG	Strong	Homozygous null mutation of Foxj1 results in complete absence of cilia in mouse respiratory epithelium Chen et al. (1998); Brody et al. (2000). In a previous study, wild-type mice had approximately 20% heavily ciliated cells in the proximal pulmonary epithelium, while explanted Foxj1^-/-^ mouse trachea had no ciliated cells Gomperts et al. (2004). Loss of FOXJ1 orthologs FoxJ1–4 in flatworm *Schmidtea mediterranea* results in loss of ciliation of the ventral epithelium which closely resembles the human airway epithelium Rompolas et al. (2009); Vij et al. (2012). Loss of Foxj1 activity in *Xenopus* and zebrafish—through antisense morpholino oligonucleotides—reduces cilia formation, while, conversely, ectopic Foxj1 overexpression results in formation of multiple motile cilia Stubbs et al. (2008); Yu et al. (2008). There is a strong correlation between FOXJ1 and expression of the FOXJ1 ciliogenesis program genes in zebrafish, *Xenopus* and mouse cells Abedalthagafi et al. (2016)
Decreased FOXJ1 protein leading to decreased motile cilia length/number		Treatment with cigarette smoke extract downregulates FOXJ1 mRNA and protein expression, which is accompanied by a reduction in cilia length and number in human bronchial epithelial cells *in vitro* Milara et al. (2012); Brekman et al. (2014). This can be prevented by overexpression of FOXJ1 Brekman et al. (2014) or treatment with roflumilast N-oxide, which reduces intracellular free radical levels and increases FOXJ1 mRNA and protein expression Milara et al. (2012)
KERH	Moderate	In *Chlamydomonas*, ciliary motion is directly related to the length of the cilia Bottier et al. (2019). Similar observations have been made in zebrafish, where modulation of cilia length and number by FOR20 (centrosomal protein 20) deletion/knockdown directly impairs ciliary motility Xie et al. (2019). There is also a positive correlation between cilia number and CBF in sinusitis patients Joki et al. (1998), while cilia number, length and orientation correlate positively with mucociliary transport rate in patients with recurrent or longstanding respiratory infections Toskala et al. (1995); Joki et al. (1998). Comparisons of strips of normal and disrupted ciliated epithelium have shown that CBF is decreased in the latter Thomas et al. (2009)
Decreased motile cilia length/number leading to decreased CBF		Mathematical models and simulations have shown that periciliary liquid and mucus velocity are directly affected by cilia number and length Lee et al. (2011); Jayathilake et al. (2012); Jayathilake et al. (2015)
KERI	Moderate	The phenomenon of ASL volume changes determining mucus viscosity is well described in the cystic fibrosis literature. In patients with this genetic defect, impaired CFTR function results in ASL depletion and mucus hyperviscosity Knowles and Boucher, (2002); Puchelle et al. (2002); Mall et al. (2004); Tarran, (2004). This has been confirmed experimentally in pig and rat models of this disease Birket et al. (2014); Birket et al. (2016); Birket et al. (2018). Studies with transgenic mice overexpressing βENaC in the airways also corroborate the link between ASL dehydration and increased mucus viscosity, evidenced by the increased incidence of airway mucus plugging [129, 195]. In a ferret model of cigarette smoke-induced COPD, ASL depletion was shown to be one of the drivers of increased mucus viscosity and decreased MCC Lin et al. (2020). The same study also showed that mucus from COPD patients, obtained from 3D organotypic airway epithelial cultures from different smoking donors with COPD, is significantly more viscous than that from healthy, non-smoking individuals and smokers without disease Lin et al. (2020)
Decreased ASL height leading to increased mucus viscosity		
KERJ	Strong	Experimental studies *in vitro* have shown that exposure of ciliated respiratory cells directly or indirectly to sources of oxidative stress leads to decreased CBF Burman and Martin, (1986); Wilson et al. (1987); Feldman et al. (1994); Yoshitsugu et al. (1995); Min et al. (1999), which can be reversed by treatment with antioxidants Schmid et al. (2015). Cigarette smoke condensate, a known inducer of oxidative stress, also causes a decrease in CBF *in vitro* Cohen et al. (2009), while, in human subjects exposed to different oxygen levels, oxygen stress causes a decrease in nasal CBF Stanek et al. (1998)
Oxidative stress leading to decreased CBF		
KERK	Moderate	Several studies have shown that there is an optimal range of viscoelastic mucus properties that facilitates efficient MCC and that changes in mucus viscosity beyond that optimal range impact CBF and alter MCC. Studies in humans, mice, hamsters, horses and frogs have shown that increased mucus viscosity correlates with a decrease in CBF King, (1979); Gheber et al. (1998); Matsui et al. (1998); Andrade et al. (2005); González et al. (2016); Kikuchi et al. (2017); Birket et al. (2018)
Increased mucus viscosity leading to decreased CBF		
KERL	Moderate	Mucus viscoelastic properties, whether altered by airway dehydration or mucus hypersecretion, directly influence MCC. Studies cystic fibrosis models and those on mimicking changes in mucus viscosity by using (bio)polymers or large molecules such as dextran have indicated a dose-response effect of increasing mucus viscosity on mucociliary transport rates, although these changes are transient in nature in *ex vivo* and *in vitro* systems Birket et al. (2018); Fernandez-Petty et al. (2019). Increased mucus viscosity also has a negative impact on MCC in horses with recurrent airway obstruction Gerber et al. (2000). Conversely, inhalation of hypertonic saline solution decreases mucus viscosity and enhances MCC in cystic fibrosis patients Robinson et al. (1997)
Increased mucus viscosity leading to decreased MCC		

Abbreviations: 3D, three-dimensional; ASL, airway surface liquid; CBF, ciliary beating frequency; CFTR, cystic fibrosis transmembrane regulator; Cl^−^, chloride (ion); COPD, chronic obstructive pulmonary disease; ENaC, epithelial sodium channel; FEF25-75, forced expiratory flow between 25 and 75% of FVC; FEV1, forced expiratory volume in 1 s; FOR20, centromere protein 20; FOXJ1, forkhead box J1; FVC, forced vital capacity; MCC, mucociliary clearance; MCT, mucociliary transport.

Exposure to inhaled oxidants, such as cigarette smoke and ozone, leads to decreased CFTR gene and protein expression as well as CFTR internalization (KE1), thereby reducing or abolishing open probabilities, short-circuit currents and subsequently ASL height/volume (KE2) (Kulka et al., 2005; Cantin et al., 2006a; Cantin et al., 2006b; Qu et al., 2009; Clunes et al., 2012; Sloane et al., 2012; Rasmussen et al., 2014). Both reduced mRNA stability (Cantin et al., 2006a) and decreased transcription rates (Bargon et al., 1992a; Bargon et al., 1992b; Rasmussen et al., 2014) reportedly contribute to diminished *CFTR* mRNA expression. CFTR expression was also modulated by STAT1 (Kulka et al., 2005; Qu et al., 2009) and Nrf2 signaling (Zhang et al., 2015). Additionally, on the post-transcriptional level, CFTR function has been shown to be affected by oxidative stress (Clunes et al., 2012) and ischemia (Brézillon et al., 1997; Bodas et al., 2017).

Serous and glandular secretions of the airway epithelium contribute to the ASL, and epithelial ion channel function is critical to ASL homeostasis. Absorption of liquid to and from the mucus layer serves to maintain ASL depth. The regulation of these reabsorption processes is complex and not fully elucidated (Boucher, 2004). Experimental evidence suggests that the balance between Na^+^ absorption and Cl^−^ secretion—mediated by ENaC and CFTR, respectively—plays a major role in these processed, with the ion channels affecting each other’s activity (Boucher, 2003; Boucher, 2004; Schmid et al., 2011). Impaired functioning of the CFTR and ENaC ion channels results in enhanced Na^+^ absorption, reduced Cl^−^ secretion, and consequently, reduced ASL height (KE2). This phenomenon is well known not only from studies in models of cystic fibrosis and acquired CFTR deficiency—even though the exact mechanism of the interaction between these two channels remains to be elucidated (Tarran et al., 2001; Boucher, 2003; Zhang et al., 2013; Hassan et al., 2014; Rasmussen et al., 2014; Woodworth, 2015; Raju et al., 2016)—but also from studies with pharmacological agents that enhance CFTR expression and/or function or perturb the interaction between CFTR and ENaC (Van Goor et al., 2009; Van Goor et al., 2011; Lambert et al., 2014).

Under physiological conditions, ASL height is adjusted to the appropriate height, which helps maintain the PCL depth at approximately the length of the cilia (Antunes and Cohen, 2007). If the airways become “dehydrated” (i.e., the ASL height decreases; KE2), the cilia collapse and ciliary movement is slowed or inhibited (KE6) (Matsui et al., 1998; Tarran et al., 2001; Knowles and Boucher, 2002; Munkholm and Mortensen, 2014). Decreased ASL height also contributes to increased mucus viscosity (KE5), a phenomenon that is well described in cystic fibrosis, where *CFTR* defect results in decreased ASL height, leading to decreased MCC (KE7) and subsequent mucus plugging (Birket et al., 2014; Birket et al., 2016; Birket et al., 2018).

Free radicals such as super oxides, hydroxyl radicals, and hydrogen peroxides are a common factor in various respiratory diseases, such as acute respiratory distress syndrome, asthma and pneumonia. Oxidative stress (such as that caused by cigarette smoke exposure or irradiation) leads to decreased FOXJ1 gene and protein expression (KE3) as well as to decreased FOXJ1 target gene expression (Milara et al., 2012; Brekman et al., 2014; Garcia-Arcos et al., 2016; Valencia-Gattas et al., 2016; Ishikawa and Ito, 2017). Because FOXJ1 is a key factor of multiple motile cilia assembly in the respiratory airways (Zhou and Roy, 2015), oxidative stress blocks the multiciliogenesis program, which is necessary and also sufficient to program cells to grow functional motile cilia (Hua et al., 2010; Vij et al., 2012). Studies in different model organisms have shown that the loss of FOXJ1 (KE3) results in a loss of multiple motile cilia (KE4) (Chen et al., 1998; Brody et al., 2000; Stubbs et al., 2008; Vij et al., 2012).

Cilia in the respiratory epithelium beat in a coordinated fashion at a frequency of approximately 7–16 Hz, propelling mucus upwards (Joki et al., 1998; Smith et al., 2012; Jing et al., 2017). Many factors have been shown to affect ciliary function, including cilia length, number, structure, orientation, and distribution as well as mucus viscosity, temperature, pH, chemicals, ASL height, and exposure to bacterial and viral pathogens (Kanthakumar et al., 1996; Clary-Meinesz et al., 1998; Joki et al., 1998; Ho et al., 2001; Mall, 2008; Smith et al., 2012; Jing et al., 2017; Snyder et al., 2017). Alterations in normal physiological conditions and healthy cilia number/length/structure (KE4) as well as oxidative stress through exposure to hydrogen peroxide or free radicals typically reduce CBF (KE6) (Burman and Martin, 1986; Clary-Meinesz et al., 1998; Min et al., 1999; Jayathilake et al., 2012).Synchronized ciliary action helps transport mucus from the lungs to the mouth, where it is swallowed or expectorated (Munkholm and Mortensen, 2014). In addition to ASL and mucus properties, the speed of mucus movement—and hence the effectiveness of MCC—is dependent on ciliary amplitude and beat frequency (Rubin, 2002). Aside from genetic defects leading to ciliopathies, there is ample evidence that prolonged exposure to noxious agents, such as cigarette smoke, nitrogen oxide and sulfur dioxide, causes a decrease in CBF (KE6) and, subsequently, MCC (KE7) (Knorst et al., 1994; Yeates et al., 1997; Kakinoki et al., 1998; Cohen et al., 2009; Schmid et al., 2015). CBF also seems to be dependent on mucus viscosity, with the CBF decreasing with increasing viscosity in animal models (Andrade et al., 2005; Kikuchi et al., 2017). This linear correlation between CBF (KE7) and mucus viscosity (KE5) has also been confirmed in mathematical models simulating the two-layer mucociliary transport process (Lee et al., 2011; Sedaghat et al., 2016).

Finally, the link between decreased MCC and decreased lung function (AO) is well established through observations in patients with ciliary defects (e.g., primary ciliary dyskinesia) and cystic fibrosis. Failure to clear mucus from the lungs causes mucus build up, which can lead to mucus plugging in the airways and, consequently, leads to decreased lung function over time (Mossberg et al., 1978; Regnis et al., 1994; Wanner et al., 1996; Robinson and Bye, 2002; Kerem et al., 2014; Szczesniak et al., 2017). Mucus plugging due to decreased MCC is also considered a major cause of airway obstruction and airflow limitation in COPD patients (Okajima et al., 2020; Dunican et al., 2021) and asthmatics (Maxwell, 1985; Kuyper et al., 2003).

## 4 Overall Assessment of the Adverse Outcome Pathways

### 4.1 Key Event Essentiality

The definition of essentiality implies that modulation of upstream KEs impacts the downstream KEs in an expected fashion. When blocked or when they fail to occur, the KEs in the current AOP will not necessarily stop the progression to subsequent KEs. Owing to the complex biology of motile cilia formation and function, ASL homeostasis, mucus properties, and MCC, the KEs and AO may be triggered because of alternative pathways or biological redundancies. However, when exacerbated, the KEs promote the occurrence of downstream events that eventually lead to the AO. The causal pathway starting from exposure to oxidants and leading to decreased lung function involves parallel routes with KEs, each of which is sufficient to cause the downstream KE to occur. Different mechanisms—such as oxidant-induced decreases in ASL height due to CFTR function decline or oxidant-induced decrease in cilia number and length as a result of decreased FOXJ1 levels—lead to decreased CBF and decreased MCC. Each of these pathways contributes to the AO, but their relative contributions are difficult to evaluate. We judge the KEs MIE, KE1, KE3, KE4, KE6, and KE7 as highly essential and suggest moderate essentiality for KE2 and KE5 (Table 1; AOPwiki, https://aopwiki.org/aops/411, https://aopwiki.org/aops/424, https://aopwiki.org/aops/425).

### 4.2 Key Event Relationship Biological Plausibility

Mechanistic data on the pathways that contribute to oxidative stress-elicited lung damage have varied coverage in current literature. The AOP network we present here starts with an oxidant exposure or exposure-causing oxidative stress leading to decreased CFTR and FOXJ1 mRNA and protein levels as well as decreased protein function. KERA (oxidative stress leading to decreased CFTR function) is supported by multiple studies across different species, which suggest their high biological plausibility (for empirical evidence supporting each KER, refer to Table 1). For a similar inhibitory role of oxidative stress on FOXJ1, the studies are less ample. However, there is credible evidence that oxidative stress has a deteriorating effect on FOXJ1 transcript and protein levels as well as on the function of this transcription factor. Therefore, we judge the plausibility of KERF (oxidative stress leading to decreased FOXJ1 protein) to be moderate.

The biological functions of CFTR and FOXJ1 are extensively studied and established across different test systems, implying the high biological plausibility of both KERB (decreased CFTR function leading to decreased ASL height) and KERG (decreased FOXJ1 protein leading to decreased motile cilia length/number). Specifically, CFTR contributes to healthy lung function by regulating epithelial ion conductance to support ASL height maintenance (Boucher, 2003; Csanady et al., 2019), and FOXJ1 is an essential factor for functional multiple motile cilia assembly (Vij et al., 2012; Choksi et al., 2014). Both decreased ASL height (KE2) and decreased motile cilia length/number (KE4) lead to decreased CBF (KE6), as outlined in KERC and KERH, respectively. Multiple studies describe the link between decreased ASL height and reduced CBF. However, the causality between these KEs is not well-established, prompting us to judge KERC as weakly supported. As for KERH, higher numbers of motile cilia with a healthy length support efficient ciliary beating, and a decrease in cilia number and/or length results in a proportionate reduction in CBF. This causal relationship is logical but is directly tested only in few studies. Therefore, we rank the biological plausibility of KERH as moderate. ASL height is also linked to the physical properties of mucus, and studies in models of or individuals with cystic fibrosis support the link between ASL depletion and increased mucus viscosity (KERI), though the overall evidence is sparse, and causality is not always proven. Because the dependencies between these two KEs were highlighted in different species *in vitro* and *in vivo*, and the underlying mechanism is well established, we judge the plausibility of KERI as moderate.

Additionally, we propose a direct relationship between oxidative stress and KE6, decreased CBF (KERJ). A variety of oxidants, such as hydrogen peroxide, nitric dioxide, sulfur dioxide, acetaldehyde, ozone, and cigarette smoke decrease CBF in airway epithelial cells in a dose- and time-dependent manner after exposure. This link is demonstrated in several studies in various species, and we judge the plausibility of KERJ as strong. Synchronized ciliary beating helps transport mucus from the distal airways to the mouth, where it is cleared through ingestion or expectoration. *In vivo* studies and observations in patients with ciliopathies, respiratory infections, or allergies, and following exposure to inhaled toxicants that compromise ciliary function demonstrate that absent, decreased, or asynchronous cilia beating results in defective mucus clearance. Pharmacological studies have demonstrated that CBF stimulation typically results in MCC stimulation. While some results support both a dose-dependent response and temporal sequence of decreased CBF (KE6) leading to decreased MCC (KE7), most studies evaluate these KEs in parallel, and no clear causal linkage is affirmed. The same is true for increased mucus viscosity. Mucus viscoelastic properties, whether altered by airway dehydration or mucus hypersecretion, directly influence MCC. In fact, there is an inverse relationship between mucus viscosity and CBF (KERK) and mucus transport/MCC (KERL), as demonstrated in several *in vivo* and *ex vivo* studies. A large proportion of these studies have employed (bio)polymers or other large organic molecules to mimic the mucus layer in the airways and the increase in its viscosity. In addition, some of these studies have shown that decreased mucus viscosity may also result in impairment of MCC. Therefore, a causal link is only tentatively supported. Because cilia function, ASL height, and mucus properties are intricately linked to each other as evidenced by cystic fibrosis studies, we consider the plausibility of KERD, KERK, and KERL as moderate.

Different routes lead to impaired MCC, such as smoking-related oxidative stress, ciliary defects or CFTR mutations. Regardless of the route that leads to a reduction in MCC, individuals with impaired MCC exhibit decreased lung function. Moreover, many pharmacological treatments that enhance MCC also improve lung function. KE7 and the AO are thus closely related; however, as causal evidence is not always available, we judge the biological plausibility of KERE (decreased MCC leading to decreased lung function) as moderate.

The linear AOPs presented here have certain knowledge gaps; however, overall, we consider the biological plausibility of our AOP network as strong, as the network integrates different plausible pathways from the same MIE, leading to a common AO. For example, while oxidative stress leading to decreased lung function through the branch MIE → KE1 → KE2 → KE5 and/or KE6 → KE7 → AO has a weakly supported link represented by KERC (KE2 → KE6, i.e., decreased ASL height leading to decreased CBF), the oxidative stress can lead to the AO also through MIE → KE6 → KE7 → AO or via decreased FOXJ1 protein levels through MIE → KE3 → KE4→ KE5 and/or KE6 → KE7 → AO.

## 5 Discussion and Conclusion

Here, we have presented an AOP that links oxidative stress resulting from inhalation exposure to toxicants to impaired lung function via a decrease in MCC. Given the individual and public health burden of the consequences of lung function impairment, gaining a greater understanding of the underlying mechanisms of this pathology is extremely important in the risk assessment of inhaled toxic chemicals. There is strong empirical evidence to support several of the KERs in this AOP, particularly at the cellular level (i.e., oxidative stress leading to decreased CFTR function [KERA], decreased CFTR function leading to decreased ASL height [KERB], and oxidative stress leading to decreased CBF [KERJ]). However, additional evidence on causality is required to attribute stronger plausibility to KERs such as that between decreased ASL height and decreased CBF (KERC), which we evaluated as being weak. Future studies, using *in vitro* assays such as those outlined in Supplementary Table S1, that directly assess this linkage as well as the KERs we determined to have moderate plausibility (i.e., KERD, KERE, KERF, KERH, KERI, KERK, and KERL) will help greatly strengthen this AOP overall.

An integrated assessment of substances with the potential to be inhaled, either intentionally or unintentionally, could incorporate inhalation exposure and dosimetry modelling to inform an *in vitro* assessment approach with appropriate exposure techniques and cell systems for assessing the KEs in this AOP (EPA’s Office of Chemical Safety and Pollution Prevention, 2019). Standardization and robustness testing of assays against explicit performance criteria using suitable reference materials can greatly increase the level of confidence in their use for KE assessment (Petersen EJ. et al., 2021; Petersen E. J. et al., 2021). Much of the empirical evidence that supports the KERs in the qualitative AOP described here was obtained from *in vitro* studies using well-established methodologies for biological endpoint assessment (Supplementary Table S1). Being chemical-agnostic, this AOP can be applied to a variety of substances that share the AO. For example, impaired MCC and decreased lung function have a long-known relationship with smoking, but little is known about the consequences of the long-term use of alternative inhaled nicotine delivery products such as electronic cigarettes and heated tobacco products. This AOP can form the basis of an assessment strategy for evaluating the effects of exposure to aerosol from these products on the basis of the KEs identified here.

AOPs such as this one can play a central role in risk assessment strategies for a wide variety of regulatory purposes by providing mechanistic support to an integrated approach to testing and assessment (IATA; (Clippinger et al., 2018)) or defined approach (DA). IATAs are flexible frameworks that can be adapted to best address the regulatory question or purpose at hand. Unlike the assessment process within IATA that involves some level of expert judgement, DA uses rule-based fixed data interpretation procedure. Both DA and IATAs are a means to integrate existing data on a chemical (e.g., physicochemical properties and ADME [absorption, distribution, metabolism, and excretion] information) with an AOP-based *in vitro* testing strategy to generate data that does not currently exist (Willett, 2019). An important feature of these approaches is that they should also include a measure of uncertainty to facilitate regulatory decision-making. AOPs can be used in an iterative fashion to identify and reduce or resolve, where possible, areas of uncertainty by generating data to fill those knowledge gaps. Furthermore, a quantitative AOP could provide data that would be translated to prediction models for human risk assessment through the application of *in vitro* to *in vivo* extrapolation (IVIVE) approaches.

## Data Availability

Publicly available datasets were analyzed in this study. This data can be found here: At the point of submission, evidence has been gathered from the literature and compiled prior to populating the AOPwiki at https://aopwiki.org/aops/411, https://aopwiki.org/aops/424 and https://aopwiki.org/aops/425. Special care was taken to properly cite all original studies that were evaluated during AOP development and assessment.

## Footnotes

1 The literature interchangeably refers to ASL height, ASL volume and ASL depth. The data presented in original research articles indicate that these terms are synonymous. To simplify the presentation of evidence, we will refer to “ASL height” here.
