# Supplementary material for: An Adverse Outcome Pathway for Decreased Lung Function Focusing on Mechanisms of Impaired Mucociliary Clearance Following Inhalation Exposure
Source: Front Toxicol. 2021 Dec 14;3:750254. doi: 10.3389/ftox.2021.750254 (PMC8915806; doi:10.3389/ftox.2021.750254)
Supplement: Supplementary file 1 [file Table1.docx]

**Supporting Information**

**An Adverse Outcome Pathway (AOP) for Decreased Lung Function Focusing on Mechanisms of Impaired Mucociliary Clearance (MCC) Following Inhalation Exposure**

**Karsta Luettich^1^, Monita Sharma^2^, Hasmik Yepiskoposyan**^1^**, Damien Breheny^3^, Frazer J. Lowe^4^**

^1^Philip Morris International R&D, Philip Morris Products S.A., Neuchatel, Switzerland

**^2^**PETA Science Consortium International e.V., Stuttgart, Germany

**^3^**British American Tobacco (Investments) Ltd., Group Research and Development, Southampton, United Kingdom

**^4^**Broughton Nicotine Services, Earby, Lancashire, United Kingdom

**Table S1.** Table of key events and corresponding *in vitro* methods that can be used for measurement of associated biological endpoints.

| Key events (KE) | | In vitro method for measurement | Reference | |
| --- | --- | --- | --- | --- |
| *MIE: Oxidative stress* | - Measurement of free radical species by electron spin resonance or radical spin trapping - Assessment of intracellular ROS load by using fluorescent probes (e.g., dihydroethidium (DHE), dichlorofluorescin diacetate (DCFDA) or dihydrorhodamine (DHR)) with fluorescence microscopy, high-content imaging or flow cytometry in 2D airway epithelial cells - Assessment of cellular antioxidant capacity by glutathione (GSH) and glutathione sulfide (GSSG) or total antioxidant capacity (TAC) assay, antioxidant enzyme (e.g., superoxide dismutase, catalase) activity assays - Assessment of oxidatively modified lipids and proteins by measurement of lipid peroxidation (e.g., malondialdehyde) or protein oxidation markers (e.g., protein carbonylation, 3-nitrotyrosine) | | | (Kohen and Nyska, 2002; Palmieri and Sblendorio, 2007; Fearon and Faux, 2009; Aranda et al., 2013; Halliwell and Gutteridge, 2015) |
| *KE1: Decreased CFTR Function* | - Assessment of expression of *CFTR* mRNA and protein in cells and tissues by (quantitative) reverse transcription polymerase chain reaction (qRT-PCR), northern blot, western blot, or immunocytochemistry - Assessment of CFTR channel function by using patch-clamping, radiolabeled-tracer efflux, or Ussing chamber | | | (Farinha et al., 2004; Li et al., 2004; Norez et al., 2004; Sheppard et al., 2004) |
| *KE2: Decreased ASL Height* | - Microscopy-based methods (e.g., confocal fluorescence scanning in the vertical plane [i.e., in XZ mode], micro-optical coherence tomography, or scanning electron microscopy) in 2D cells and 3D organotypic airway epithelial models | | | (Matsui et al., 1998; Tarran et al., 2001; Tarran and Boucher, 2002; Lazarowski et al., 2004; Roomans et al., 2004; Tarran et al., 2005; Tarran et al., 2006; Garcia-Caballero et al., 2009; Saint-Criq et al., 2013; Zhang et al., 2013) (Raju et al., 2016) |
| *KE3: Decreased FOXJ1 Protein* | - Assessment of FOXJ1 protein levels by western blot analysis, immunofluorescence analysis, or immunohistochemistry - Assessment of FOXJ1 mRNA levels by qRT-PCR, *in situ* hybridization, northern blot analysis - Assessment of FOXJ1 protein activity from target gene expression levels or from reporter gene expression levels (e.g., luciferase assay) of genes harboring FOXJ1 transcription factor binding sites | | | (Hackett et al., 1995; Lim et al., 1997; Danielian et al., 2007; Gomperts et al., 2007; Jacquet et al., 2009; Milara et al., 2012; Stubbs et al., 2012; Didon et al., 2013; Brekman et al., 2014; Gao et al., 2015; Abedalthagafi et al., 2016; Arbi et al., 2016; Valencia-Gattas et al., 2016). |
| *KE4: Decreased Multiple Motile Cilia Length/Number* | - Quantification of mature cilia numbers from ciliary precursors (immunofluorescence assay, three-dimensional superresolution structured illumination microscopy [3D-SIM]) - Measurement of cilia length by using immunofluorescence followed by microscopy and quantification software (using, e.g., ImageJ software or MetaMorph Microscopy Automation & Image Analysis Software) | | | (Leopold et al., 2009; Brekman et al., 2014; Li et al., 2014; Nanjundappa et al., 2019). |
| *KE5: Increased mucus viscosity* | - Determination of mucus composition (expression of MUC mRNA and protein) in cells and tissues by qRT-PCR, northern blot, western blot, immunocytochemistry, or mass spectrometry - Assessment of mucus viscosity by microrheology, particle movement tracking through videomicroscopy or fluorescence recovery after photobleaching (FRAP) | | | (Lai et al., 2009; Liu et al., 2015; Hill et al., 2018; Lock et al., 2018; Atanasova and Reznikov, 2019; Chen et al., 2019) |
| *KE6: Decreased Ciliary Beat Frequency* | - Estimation of active area and ciliary beat frequency by photometry and video-microscopy | | | (Agius et al., 1998; Min et al., 1999; Uzlaner and Priel, 1999; Sisson et al., 2003; Dimova et al., 2005; Allen-Gipson et al., 2011; Kim et al., 2011; Feriani et al., 2017; Peabody et al., 2018) |
| *KE7: Decreased MCC* | - Determination of mucus transport speed/rate by particle movement tracking through videomicroscopy in 3D airway epithelial cultures | | | (Knowles and Boucher, 2002; Fliegauf et al., 2013; Liu et al., 2015; Sears et al., 2015; Benam et al., 2018; Hill et al., 2018) |

References

Abedalthagafi, M.S., Wu, M.P., Merrill, P.H., Du, Z., Woo, T., Sheu, S.H., et al. (2016). Decreased FOXJ1 expression and its ciliogenesis programme in aggressive ependymoma and choroid plexus tumours. *J. Pathol.* 238(4)**,** 584-597. doi: 10.1002/path.4682.

Agius, A.M., Smallman, L.A., and Pahor, A.L. (1998). Age, smoking and nasal ciliary beat frequency. *Clin. Otolaryngol. Allied Sci.* 23(3)**,** 227-230. doi: doi:10.1046/j.1365-2273.1998.00141.x.

Allen-Gipson, D.S., Blackburn, M.R., Schneider, D.J., Zhang, H., Bluitt, D.L., Jarrell, J.C., et al. (2011). Adenosine activation of A(2B) receptor(s) is essential for stimulated epithelial ciliary motility and clearance. *Am. J. Physiol. Lung Cell. Mol. Physiol.* 301(2)**,** L171-L180. doi: 10.1152/ajplung.00203.2010.

Aranda, A., Sequedo, L., Tolosa, L., Quintas, G., Burello, E., Castell, J., et al. (2013). Dichloro-dihydro-fluorescein diacetate (DCFH-DA) assay: a quantitative method for oxidative stress assessment of nanoparticle-treated cells. *Toxicol. In Vitro* 27(2)**,** 954-963.

Arbi, M., Pefani, D.E., Kyrousi, C., Lalioti, M.E., Kalogeropoulou, A., Papanastasiou, A.D., et al. (2016). GemC1 controls multiciliogenesis in the airway epithelium. *EMBO Rep.* 17(3)**,** 400-413. doi: 10.15252/embr.201540882.

Atanasova, K.R., and Reznikov, L.R. (2019). Strategies for measuring airway mucus and mucins. *Respir. Res.* 20(1)**,** 261. doi: 10.1186/s12931-019-1239-z.

Benam, K.H., Vladar, E.K., Janssen, W.J., and Evans, C.M. (2018). Mucociliary defense: emerging cellular, molecular, and animal models. *Ann. Am. Thorac. Soc.* 15(Supplement 3)**,** S210-S215.

Brekman, A., Walters, M.S., Tilley, A.E., and Crystal, R.G. (2014). FOXJ1 Prevents Cilia Growth Inhibition by Cigarette Smoke in Human Airway Epithelium In Vitro. *American Journal of Respiratory Cell and Molecular Biology* 51(5)**,** 688-700. doi: 10.1165/rcmb.2013-0363OC.

Chen, Z., Zhong, M., Luo, Y., Deng, L., Hu, Z., and Song, Y. (2019). Determination of rheology and surface tension of airway surface liquid: a review of clinical relevance and measurement techniques. *Respir. Res.* 20(1)**,** 1-14.

Danielian, P.S., Kim, C.F.B., Caron, A.M., Vasile, E., Bronson, R.T., and Lees, J.A. (2007). E2f4 is required for normal development of the airway epithelium. *Dev. Biol.* 305(2)**,** 564-576.

Didon, L., Zwick, R.K., Chao, I.W., Walters, M.S., Wang, R., Hackett, N.R., et al. (2013). RFX3 Modulation of FOXJ1 regulation of cilia genes in the human airway epithelium. *Respir. Res.* 14(1)**,** 70-70. doi: 10.1186/1465-9921-14-70.

Dimova, S., Maes, F., Brewster, M.E., Jorissen, M., Noppe, M., and Augustijns, P. (2005). High-speed digital imaging method for ciliary beat frequency measurement. *J. Pharm. Pharmacol.* 57(4)**,** 521-526. doi: doi:10.1211/0022357055777.

Farinha, C.M., Penque, D., Roxo-Rosa, M., Lukacs, G., Dormer, R., McPherson, M., et al. (2004). Biochemical methods to assess CFTR expression and membrane localization. *J. Cyst. Fibros.* 3**,** 73-77. doi: 10.1016/j.jcf.2004.05.017.

Fearon, I.M., and Faux, S.P. (2009). Oxidative stress and cardiovascular disease: novel tools give (free) radical insight. *J. Mol. Cell. Cardiol.* 47(3)**,** 372-381.

Feriani, L., Juenet, M., Fowler, C.J., Bruot, N., Chioccioli, M., Holland, S.M., et al. (2017). Assessing the Collective Dynamics of Motile Cilia in Cultures of Human Airway Cells by Multiscale DDM. *Biophys. J.* 113(1)**,** 109-119. doi: 10.1016/j.bpj.2017.05.028.

Fliegauf, M., Sonnen, A.F.P., Kremer, B., and Henneke, P. (2013). Mucociliary Clearance Defects in a Murine In Vitro Model of Pneumococcal Airway Infection. *PLoS one* 8(3)**,** e59925. doi: 10.1371/journal.pone.0059925.

Gao, X., Bali, A.S., Randell, S.H., and Hogan, B.L. (2015). GRHL2 coordinates regeneration of a polarized mucociliary epithelium from basal stem cells. *J. Cell. Biol.***,** jcb. 201506014.

Garcia-Caballero, A., Rasmussen, J.E., Gaillard, E., Watson, M.J., Olsen, J.C., Donaldson, S.H., et al. (2009). SPLUNC1 regulates airway surface liquid volume by protecting ENaC from proteolytic cleavage. *Proc. Natl. Acad. Sci. U. S. A.* 106(27)**,** 11412-11417.

Gomperts, B.N., Kim, L.J., Flaherty, S.A., and Hackett, B.P. (2007). IL-13 Regulates Cilia Loss and foxj1 Expression in Human Airway Epithelium. *Am. J. Respir. Cell Mol. Biol.* 37(3)**,** 339-346. doi: 10.1165/rcmb.2006-0400OC.

Hackett, B.P., Brody, S.L., Liang, M., Zeitz, I.D., Bruns, L.A., and Gitlin, J.D. (1995). Primary structure of hepatocyte nuclear factor/forkhead homologue 4 and characterization of gene expression in the developing respiratory and reproductive epithelium. *Proc. Natl. Acad. Sci. U. S. A.* 92(10)**,** 4249-4253.

Halliwell, B., and Gutteridge, J.M. (2015). *Free radicals in biology and medicine.* Oxford university press, USA.

Hill, D.B., Long, R.F., Kissner, W.J., Atieh, E., Garbarine, I.C., Markovetz, M.R., et al. (2018). Pathological mucus and impaired mucus clearance in cystic fibrosis patients result from increased concentration, not altered pH. *Eur. Respir. J.* 52(6).

Jacquet, B.V., Salinas-Mondragon, R., Liang, H., Therit, B., Buie, J.D., Dykstra, M., et al. (2009). FoxJ1-dependent gene expression is required for differentiation of radial glia into ependymal cells and a subset of astrocytes in the postnatal brain. *Development* 136(23)**,** 4021-4031. doi: 10.1242/dev.041129.

Kim, W., Han, T.H., Kim, H.J., Park, M.Y., Kim, K.S., and Park, R.W. (2011). An Automated Measurement of Ciliary Beating Frequency using a Combined Optical Flow and Peak Detection. *J. Healthc. Inform. Res.* 17(2)**,** 111-119. doi: 10.4258/hir.2011.17.2.111.

Knowles, M.R., and Boucher, R.C. (2002). Mucus clearance as a primary innate defense mechanism for mammalian airways. *J. Clin. Investig.* 109(5)**,** 571-577.

Kohen, R., and Nyska, A. (2002). Invited review: Oxidation of biological systems: oxidative stress phenomena, antioxidants, redox reactions, and methods for their quantification. *Toxicol. Pathol.* 30(6)**,** 620-650.

Lai, S.K., Wang, Y.-Y., Wirtz, D., and Hanes, J. (2009). Micro- and macrorheology of mucus. *Adv. Drug Deliv. Rev.* 61(2)**,** 86-100. doi: 10.1016/j.addr.2008.09.012.

Lazarowski, E.R., Tarran, R., Grubb, B.R., Van Heusden, C.A., Okada, S., and Boucher, R.C. (2004). Nucleotide release provides a mechanism for airway surface liquid homeostasis. *J. Biol. Chem.*

Leopold, P.L., O'Mahony, M.J., Lian, X.J., Tilley, A.E., Harvey, B.G., and Crystal, R.G. (2009). Smoking is associated with shortened airway cilia. *PLoS one* 4(12)**,** e8157. doi: 10.1371/journal.pone.0008157.

Li, H., Sheppard, D.N., and Hug, M.J. (2004). Transepithelial electrical measurements with the Ussing chamber. *J. Cyst. Fibros.* 3**,** 123-126. doi: 10.1016/j.jcf.2004.05.026.

Li, Y.Y., Li, C.W., Chao, S.S., Yu, F.G., Yu, X.M., Liu, J., et al. (2014). Impairment of cilia architecture and ciliogenesis in hyperplastic nasal epithelium from nasal polyps. *J. Allergy Clin. Immunol.* 134(6)**,** 1282-1292. doi: 10.1016/j.jaci.2014.07.038.

Lim, L., Zhou, H., and Costa, R.H. (1997). The winged helix transcription factor HFH-4 is expressed during choroid plexus epithelial development in the mouse embryo. *Proc. Natl. Acad. Sci. U. S. A.* 94(7)**,** 3094-3099. doi: 10.1073/pnas.94.7.3094.

Liu, M., Zhang, J., Shan, W., and Huang, Y. (2015). Developments of mucus penetrating nanoparticles. *Asian J. Pharm. Sci.* 10(4)**,** 275-282. doi: 10.1016/j.ajps.2014.12.007.

Lock, J.Y., Carlson, T.L., and Carrier, R.L. (2018). Mucus models to evaluate the diffusion of drugs and particles. *Adv. Drug Deliv. Rev.* 124**,** 34-49. doi: 10.1016/j.addr.2017.11.001.

Matsui, H., Grubb, B.R., Tarran, R., Randell, S.H., Gatzy, J.T., Davis, C.W., et al. (1998). Evidence for periciliary liquid layer depletion, not abnormal ion composition, in the pathogenesis of cystic fibrosis airways disease. *Cell* 95(7)**,** 1005-1015.

Milara, J., Armengot, M., Bañuls, P., Tenor, H., Beume, R., Artigues, E., et al. (2012). Roflumilast N-oxide, a PDE4 inhibitor, improves cilia motility and ciliated human bronchial epithelial cells compromised by cigarette smoke in vitro. *Br. J. Pharmacol.* 166(8)**,** 2243-2262. doi: 10.1111/j.1476-5381.2012.01929.x.

Min, Y.G., Ohyama, M., Lee, K.S., Rhee, C.S., Oh, S.H., Sung, M.W., et al. (1999). Effects of free radicals on ciliary movement in the human nasal epithelial cells. *Auris, nasus, larynx* 26(2)**,** 159-163.

Nanjundappa, R., Kong, D., Shim, K., Stearns, T., Brody, S.L., Loncarek, J., et al. (2019). Regulation of cilia abundance in multiciliated cells. *Elife* 8. doi: 10.7554/eLife.44039.

Norez, C., Heda, G.D., Jensen, T., Kogan, I., Hughes, L.K., Auzanneau, C., et al. (2004). Determination of CFTR chloride channel activity and pharmacology using radiotracer flux methods. *J. Cyst. Fibros.* 3**,** 119-121. doi: 10.1016/j.jcf.2004.05.025.

Palmieri, B., and Sblendorio, V. (2007). Oxidative stress tests: overview on reliability and use. *Eur. Rev. Med. Pharmacol. Sci.* 11(6)**,** 383-399.

Peabody, J.E., Shei, R.-J., Bermingham, B.M., Phillips, S.E., Turner, B., Rowe, S.M., et al. (2018). Seeing cilia: imaging modalities for ciliary motion and clinical connections. *Am. J. Physiol. Lung Cell. Mol. Physiol.* 314(6)**,** L909-L921. doi: 10.1152/ajplung.00556.2017.

Raju, S.V., Lin, V.Y., Liu, L., Mcnicholas, C.M., Karki, S., Sloane, P.A., et al. (2016). The Cftr Potentiator Ivacaftor Augments Mucociliary Clearance Abrogating Cftr Inhibition by Cigarette Smoke. *Am. J. Respir. Cell Mol. Biol.*

Roomans, G.M., Kozlova, I., Nilsson, H., Vanthanouvong, V., Button, B., and Tarran, R. (2004). Measurements of airway surface liquid height and mucus transport by fluorescence microscopy, and of ion composition by X-ray microanalysis. *J. Cyst. Fibros.* 3**,** 135-139. doi: 10.1016/j.jcf.2004.05.029.

Saint-Criq, V., Kim, S.H., Katzenellenbogen, J.A., and Harvey, B.J. (2013). Non-Genomic Estrogen Regulation of Ion Transport and Airway Surface Liquid Dynamics in Cystic Fibrosis Bronchial Epithelium. *PloS one* 8(11)**,** e78593. doi: 10.1371/journal.pone.0078593.

Sears, P.R., Yin, W.-N., and Ostrowski, L.E. (2015). Continuous mucociliary transport by primary human airway epithelial cells in vitro. *Am. J. Physiol. Lung Cell. Mol. Physiol.* 309(2)**,** L99-L108. doi: 10.1152/ajplung.00024.2015.

Sheppard, D.N., Gray, M.A., Gong, X., Sohma, Y., Kogan, I., Benos, D.J., et al. (2004). The patch-clamp and planar lipid bilayer techniques: powerful and versatile tools to investigate the CFTR Cl− channel. *J. Cyst. Fibros.* 3**,** 101-108. doi: 10.1016/j.jcf.2004.05.046.

Sisson, J.H., Stoner, J., Ammons, B., and Wyatt, T. (2003). All‐digital image capture and whole‐field analysis of ciliary beat frequency. *J. Microsc.* 211(2)**,** 103-111.

Stubbs, J.L., Vladar, E.K., Axelrod, J.D., and Kintner, C. (2012). Multicilin promotes centriole assembly and ciliogenesis during multiciliate cell differentiation. *Nat. Cell Biol.* 14(2)**,** 140-147. doi: 10.1038/ncb2406.

Tarran, R., and Boucher, R.C. (2002). "Thin-film measurements of airway surface liquid volume/composition and mucus transport rates in vitro," in *Cystic fibrosis methods and protocols*. Springer), 479-492.

Tarran, R., Button, B., Picher, M., Paradiso, A.M., Ribeiro, C.M., Lazarowski, E.R., et al. (2005). Normal and cystic fibrosis airway surface liquid homeostasis The effects of phasic shear stress and viral infections. *J. Biol. Chem.* 280(42)**,** 35751-35759.

Tarran, R., Grubb, B.R., Gatzy, J.T., Davis, C.W., and Boucher, R.C. (2001). The relative roles of passive surface forces and active ion transport in the modulation of airway surface liquid volume and composition. *J. Gen. Physiol.* 118(2)**,** 223-236.

Tarran, R., Trout, L., Donaldson, S.H., and Boucher, R.C. (2006). Soluble mediators, not cilia, determine airway surface liquid volume in normal and cystic fibrosis superficial airway epithelia. *J. Gen. Physiol.* 127(5)**,** 591-604.

Uzlaner, N., and Priel, Z. (1999). Interplay between the NO pathway and elevated [Ca(2+)](i) enhances ciliary activity in rabbit trachea. *J. Physiol.* 516(Pt 1)**,** 179-190. doi: 10.1111/j.1469-7793.1999.179aa.x.

Valencia-Gattas, M., Conner, G.E., and Fregien, N.L. (2016). Gefitinib, an EGFR Tyrosine Kinase inhibitor, Prevents Smoke-Mediated Ciliated Airway Epithelial Cell Loss and Promotes Their Recovery. *PloS one* 11(8)**,** e0160216. doi: 10.1371/journal.pone.0160216.

Zhang, S., Blount, A.C., McNicholas, C.M., Skinner, D.F., Chestnut, M., Kappes, J.C., et al. (2013). Resveratrol Enhances Airway Surface Liquid Depth in Sinonasal Epithelium by Increasing Cystic Fibrosis Transmembrane Conductance Regulator Open Probability. *PloS one* 8(11)**,** e81589. doi: 10.1371/journal.pone.0081589.
